# Right ventriculo-arterial coupling in repaired Fallot patients with pulmonary valve regurgitation before and after pulmonary valve implantation: a CMR study

**DOI:** 10.1186/1532-429X-17-S1-P200

**Published:** 2015-02-03

**Authors:** Francesca R Pluchinotta, Gianfranco Butera, Francesco Secchi, Francesco Sardanelli, Mario Carminati, Massimo Lombardi

**Affiliations:** Multimodality Cardiac Imaging Section - CMR Unit, IRCCS Policlinico San Donato, San Donato Milanese (MI), Italy; Department of Pediatric Cardiology and Adult Congenital Heart Disease, IRCCS Policlinico San Donato, San Donato Milanese (MI), Italy; Department of Radiology, IRCCS Policlinico San Donato, San Donato Milanese (MI), Italy

## Background

Right ventricular-pulmonary arterial coupling plays an important role in the occurrence of right ventricular failure. Ventricular-arterial coupling is defined as Ea/Emax. Ea is the effective arterial elastance and an index of the post-load, and includes vascular resistances, vessel compliance, vascular impedance, systolic and diastolic time intervals. Ea = (arterial end-systolic pressure/stroke volume), where arterial end-systolic pressure = (mean pulmonary arterial pressure - wedge capillary pressure). The role of wedge capillary pressure can be considered negligible in subjects with normal pulmonary artery pressure and normal left ventricular (LV) function. Therefore, the formula can be simplified to Ea = (mean pulmonary arterial pressure/stroke volume). Emax is the maximal systolic ventricular elastance. This is a load-independent index. It occurs at the end of the systole and is given by the formula Emax = [end-systolic pressure/(end-systolic volume - volume (theorical) at zero load)]. Volume at zero load can be considered negligible, and end-systolic pressure can be approximated by mean pulmonary arterial pressure. Therefore Emax = (mean pulmonary artery pressure/end-systolic volume). Consequently, Ea/Emax = [(mean pulmonary artery pressure/stroke volume)/(end-systolic volume/mean pulmonary artery pressure)] and results in Ea/Emax = (end-systolic volume/stroke volume). Optimal ventricular-arterial coupling is when Ea/Emax=1.

Objective of this study is to evaluate by CMR the right ventricular-arterial coupling before and after surgical or transcatheter pulmonary valve implantation (PVI) in subjects who underwent surgery for tetralogy of Fallot (ToF) and have pulmonary valve (PV) regurgitation and/or stenosis.

## Methods

We evaluated 34 patients (age 23 ± 21 years; BSA 1.61 ± 0.5) treated for ToF who have PV regurgitation and/or stenosis and underwent surgical or transcatheter PVI. CMR studies were performed before and 12 months after PVI. The volumes and stroke volumes were adjusted for BSA. Ventriculo-arterial coupling indexes were compared before and after PVI with paired t-test or with Wilcoxon rank-sum test as appropriate. For the evaluation of a possible influence of other variables, multivariate regression analysis was performed adjusting for age, sex, type of treatment (surgery vs transcatheter), basal and post-treatment LV EF, years from surgery). All test were two-tailed and p-value <0.05 was considered significant.

## Results

Fifteen subjects were submitted to a surgical procedure while 19 underwent a transcatheter approach. All subjects completed the pre-operative and the 12 months follow-up evaluations. The Ea/Emax changed significantly between the pre-operative and the post-operative period (pre 1.42 ± 1.06 versus post 1.02 ± 0.43; p=0.027). No relationships were found with age at procedure, age at evaluation, sex and group of treatment.

## Conclusions

In our series procedures of PVI normalize the right ventricular-pulmonary arterial coupling at 12 months of follow-up.

## Funding

N/A.

